# Hospital Networks and the Dispersal of Hospital-Acquired Pathogens by Patient Transfer

**DOI:** 10.1371/journal.pone.0035002

**Published:** 2012-04-25

**Authors:** Tjibbe Donker, Jacco Wallinga, Richard Slack, Hajo Grundmann

**Affiliations:** 1 Department of Medical Microbiology, University Medical Centre Groningen, Groningen, The Netherlands; 2 Centre for Infectious Disease Control, National Institute for Public Health and the Environment, Bilthoven, The Netherlands; 3 Julius Center for Health Research and Primary Care, University Medical Center Utrecht, Utrecht, The Netherlands; 4 Health Protection Agency, East Midlands, Nottingham, United Kingdom; Northeastern University, United States of America

## Abstract

Hospital-acquired infections (HAI) are often seen as preventable incidents that result from unsafe practices or poor hospital hygiene. This however ignores the fact that transmissibility is not only a property of the causative organisms but also of the hosts who can translocate bacteria when moving between hospitals. In an epidemiological sense, hospitals become connected through the patients they share. We here postulate that the degree of hospital connectedness crucially influences the rates of infections caused by hospital-acquired bacteria. To test this hypothesis, we mapped the movement of patients based on the UK-NHS Hospital Episode Statistics and observed that the proportion of patients admitted to a hospital after a recent episode in another hospital correlates with the hospital-specific incidence rate of MRSA bacteraemia as recorded by mandatory reporting. We observed a positive correlation between hospital connectedness and MRSA bacteraemia incidence rate that is significant for all financial years since 2001 except for 2008–09. All years combined, this correlation is positive and significantly different from zero (partial correlation coefficient r = 0.33 (0.28 to 0.38)). When comparing the referral pattern for English hospitals with referral patterns observed in the Netherlands, we predict that English hospitals more likely see a swifter and more sustained spread of HAIs. Our results indicate that hospitals cannot be viewed as individual units but rather should be viewed as connected elements of larger modular networks. Our findings stress the importance of cooperative effects that will have a bearing on the planning of health care systems, patient management and hospital infection control.

## Introduction

The spread of hospital-acquired infections (HAI) has been mainly studied at the level of single hospitals [1], [2], as most investigators have focussed on the immediate causes of nosocomial transmission [3], [4]. These causes consist of a mix of risk factors which are pathogen–, patient– and health care-related [5]. HAIs are mainly caused by opportunistic bacteria often belonging to successful clonal lineages [6], [7] with frequent resistance to antibiotics, which enhances their dispersal ability in settings where vulnerable patients receive multiple antibiotic therapies [8]. Health care-related risk factors are of particular importance as they offer the most tangible explanations, and there is a body of evidence that relate poor hygienic standards or unsafe practices with increased rates of HAIs [5], [9]–[11]. The inverse relation between infection control and infection rate provides a compelling basis for the benchmarking of hospitals using indicator pathogens such as methicillin-resistant *Staphylococcus aureus* (MRSA) and *Clostridium difficile* as indirect measures of performance of infection control.

Beyond the observational unit of single hospitals, there is another layer that has remained largely unexplored during the recent scientific discourse. Hospitals refer patients for numerous reasons to other hospitals and these patients may translocate hospital-acquired pathogens between health care institutions. From an epidemiological point of view, hospitals become connected through their shared patients [12]–[15]. By tracking all admissions and discharges in a country over time, the structure of the national hospital referral network can be revealed.

We here suggest patient movement between hospitals as an alternative, more parsimonious explanation for the variation in the incidence of HAIs caused by hospital-acquired bacteria (such as MRSA, carbapenemase-producing *Klebsiella pneumoniae*, or *C. difficile*) at single hospital, regional and national level. We explored this hypothesis by quantifying patient movements between health care institutions using a social network approach. To that effect, we reconstructed the national hospital referral network for England, based on all annual patient admissions recorded by the National Health Service (NHS) Hospital Episode Statistic. In this way, we were able to test if the network effects of patient referrals can explain hospital-specific incidence rates and historical trends reported by the Department of Health's mandatory surveillance of MRSA bacteraemia for English hospital trusts. Furthermore, we extended our network analysis by comparing hospital utilisation between England and the Netherlands in order to test if differences in patient referrals contribute to the discrepancies in MRSA prevalence observed in both countries and to what degree health care systems facilitate the nation-wide dispersal of hospital-acquired pathogens.

## Results

We reconstructed the English hospital referral network for financial year (FY) 2006–07 (April 2006 – March 2007, figure 1), based on individual patient admission and discharge data, registered in the NHS Hospital Episode Statistics. Subsequently, we inferred characteristics of this referral network and correlated those with the MRSA bacteraemia incidence rate as reported in the English mandatory MRSA surveillance scheme from FY 2001–02 to FY 2008–09. We weighted the connections between all hospitals by the rate at which patients can displace pathogens between them. Although many hospitals are interconnected, only few are connected through very strong links; eliminating 95% of the weakest links reduced the total overall connectivity by only 15%. (For a detailed description of the network properties see text S1 and figure S1).

**Figure 1:**
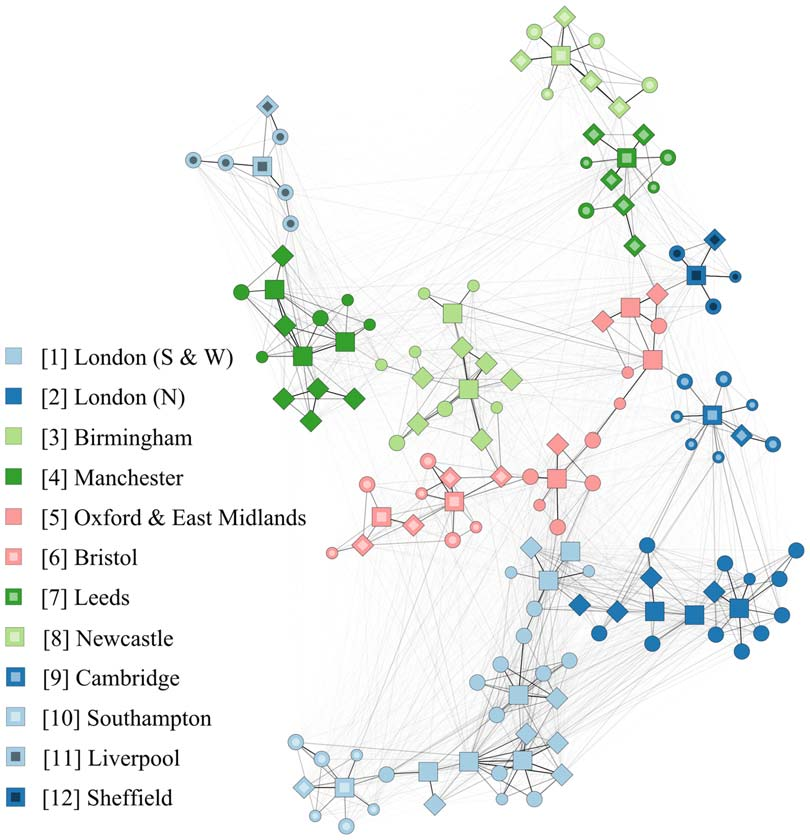
The structure of the hospital referral network in England, based on the NHS Hospital Episode Statistics for the year 2006–07. In this period, 7,420,219 patients were admitted to 146 acute NHS hospital trusts, for a total of 12,929,171 health-care episodes (corresponding to 143 inpatients and 249 admissions per 1000 inhabitants). Markers indicate hospitals; squares, diamonds, large dots and small dots denote respectively the acute teaching, large, medium and small acute hospitals. The thickness of the lines between nodes indicates the number of patients that are referred between hospitals. Different colours indicate regional hospital clusters as identified by community detection algorithm and defined as hospitals that share more patients among themselves than with other hospitals. Typically, regional hospital clusters are centred around acute teaching hospitals, and have a total number of hospitals ranging from 5 (in Sheffield) to 25 hospitals (London South & West). Hospital clusters are numbered according to size.

**Figure 2:**
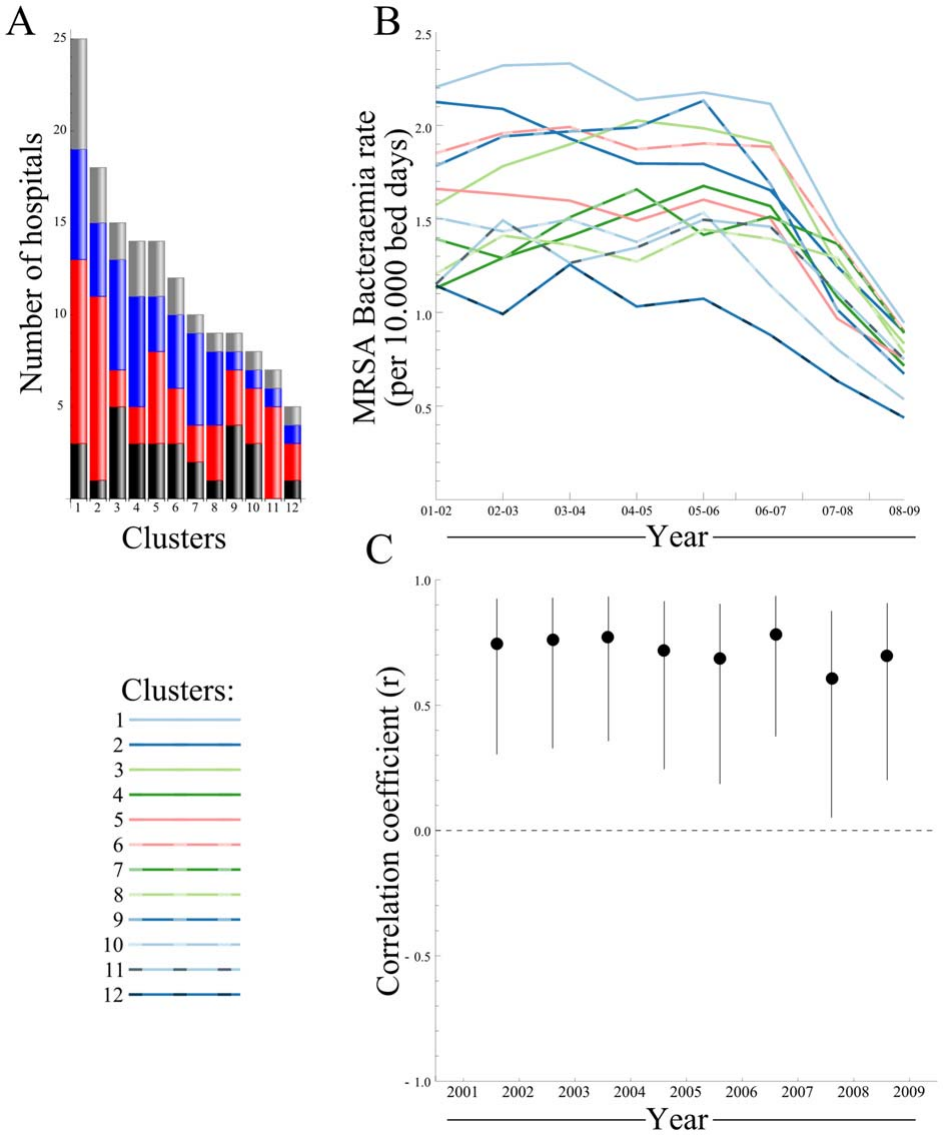
Differences between hospital clusters in the English hospital referral network. A) The size distribution and composition of hospital clusters, categories are acute teaching (gray), large (blue), medium (red) and small acute hospitals (black). B) The MRSA bacteraemia incidence rate per hospital cluster, between 2001 and 2009, although the overall MRSA bacteraemia incidence rate declines, the ordering of clusters remains largely the same; London S&W (cluster 1) presents highest rates in all years, whereas Sheffield (cluster 12) shows lowest rates in all years except 2001–02 C) The correlation between the number of hospitals within a cluster and the mean incidence rate is significant in all years. Larger clusters show higher rates in all years.

Apart from a high degree of connectedness, the English referral network is highly structured and can be subdivided into 12 hospital regional clusters (figure 1 and 2 A), captured in the network community structure [16]. Clustered hospitals have strong links among themselves, whereby more patients are exchanged between hospitals within the clusters than with hospitals outside the cluster. The consensus partitioning into the 12 hospital regional clusters was supported by 5,000 bootstrap simulations (average bootstrap value of 97.6%) and showed high modularity (See table S1), revealing a hierarchical modular network architecture. Hospital regional clusters ranged in size between 5 (Sheffield) and 25 (London South & West) hospitals. We demonstrated a consistent positive association between the number of hospitals within each cluster and their mean MRSA incidence rate for the entire reporting period (figure 2 B and C).

**Figure 3:**
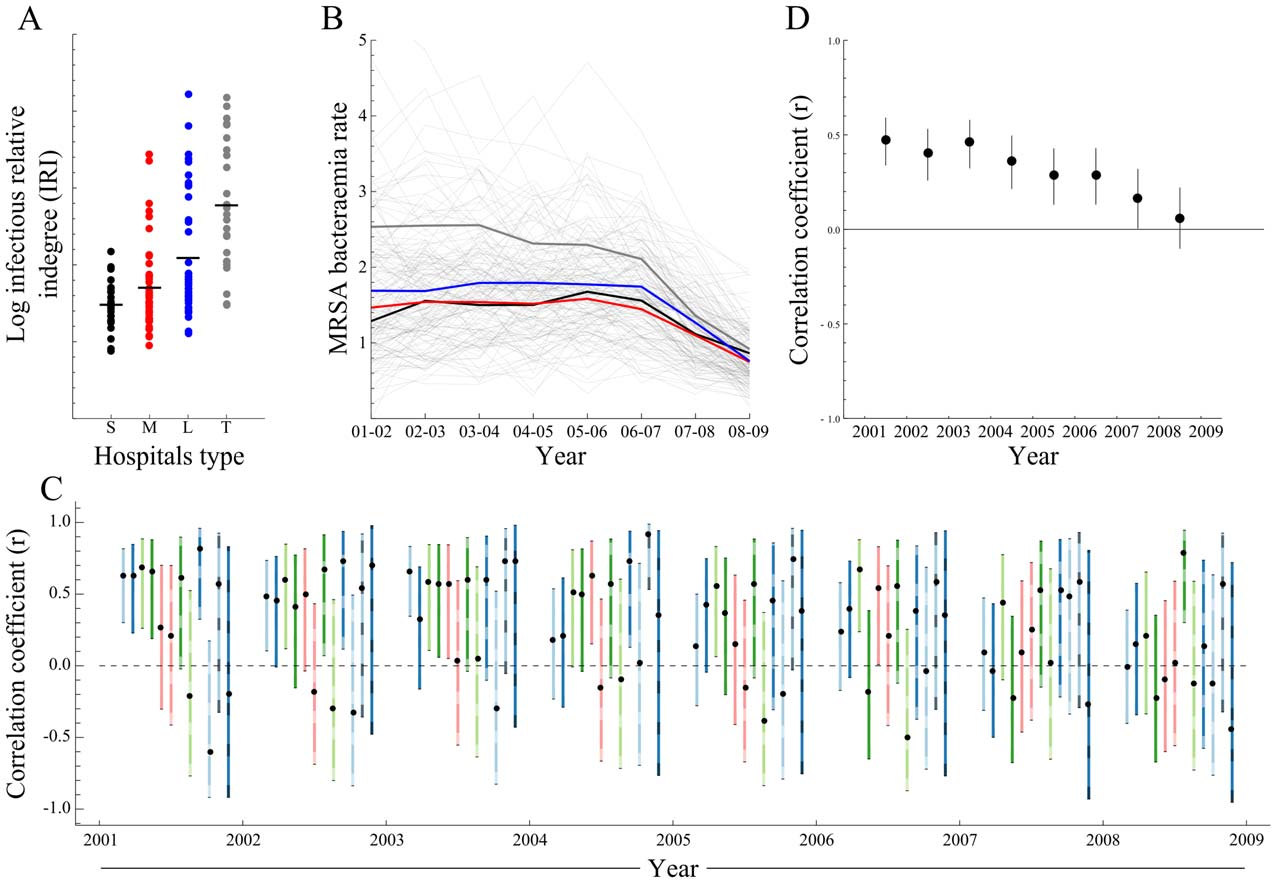
Correlation between proportion of potentially infectious patients among all admitted patients (infectious relative indegree, IRI) and the MRSA bacteraemia incidence rate at hospital level, in England between 2001 and 2009. A) The proportion of potentially infectious patients among all admitted patients (log IRI) by hospital category. This proportion increases with hospital category, from small acute care hospitals to teaching hospitals. B) The MRSA bacteraemia incidence rate per hospital, between 2001 and 2009 (thin lines), and the mean per hospital category (Thick Lines), the MRSA incidence rate is highest in acute teaching hospitals. C) Correlation between the hospital log IRI and MRSA bacteraemia incidence rate for all regional hospital clusters. Over the 8 years, 20 times a cluster showed a significant positive correlation, while none showed a significant negative correlation. D) Partial correlation coefficient between the hospital log IRI and MRSA bacteraemia incidence rate for all hospitals, adjusted for incidence differences of regional clusters. Hospitals with a high degree of connectedness show higher MRSA rates than their lesser connected counterparts.

In order to explore the extent to which patients could possibly translocate hospital-acquired pathogens, the infectious relative indegree (IRI) was recorded for each hospital. IRI is defined as the number of potentially infectious patients a hospital receives through referrals from other hospitals, divided by the hospital's total number of admissions. Average IRI increases with hospital category, which can be small, medium, large or teaching (figure 3 A), related to the different functions these categories of hospitals have in the health care system. An analogous pattern can be observed in the mean MRSA bacteraemia incidence rates reported by hospital category between FY 2001–02 and 2008–09 (figure 3 B).

When testing for correlation between hospital-specific IRIs and MRSA bacteraemia incidence rates for each of the 12 clusters during a period of eight years (FY 2001–02 to 2008–09), the majority of cluster-years (76%, 73/96) showed a positive correlation of which over 27% (20/73) were significant, and a minority of cluster-years (24%, 23/96) showed a negative correlation of which none reached statistical significance (figure 3 C). It thus appears that the MRSA incidence rate of an individual hospital is contingent on the number of patients it shares with other hospitals within its cluster.

We calculated the partial correlation coefficient for all hospitals, adjusted for cluster-specific mean MRSA bacteraemia rates, and observed a positive correlation between IRI and MRSA bacteraemia incidence rate that is significant for all financial years except for the last (FY 2008–09, figure 3 D). All years combined, this correlation is positive and significantly different from zero (partial correlation coefficient r = 0.33, 95% CI 0.28 to 0.38). Overall, strongly connected hospitals thus have significant higher MRSA bacteraemia rates than the less connected institutions.

**Figure 4:**
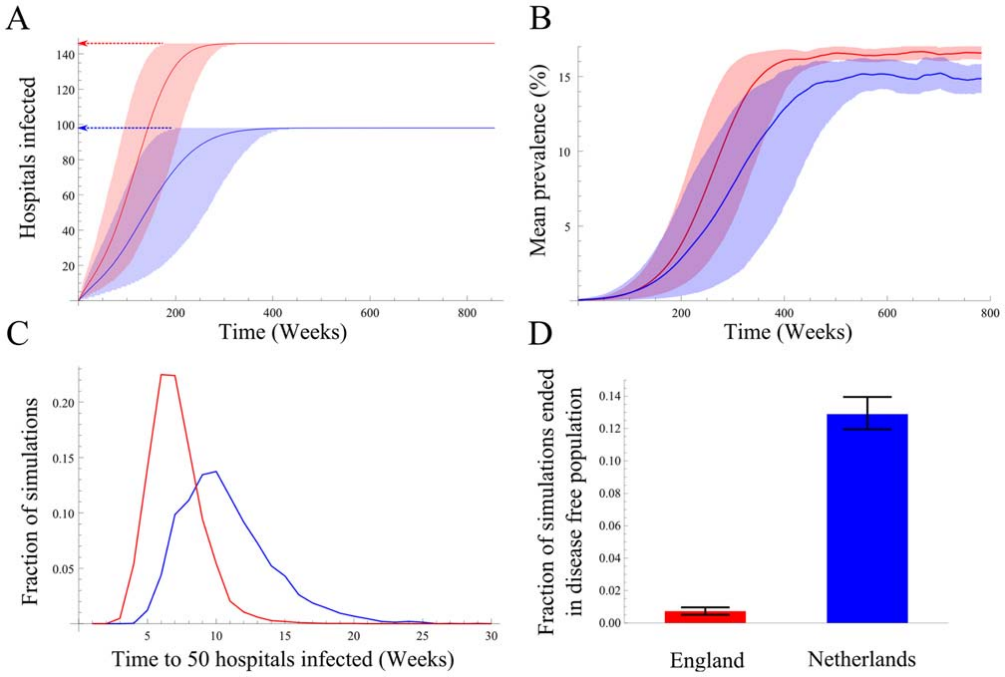
The simulated spread of MRSA at national level (assuming no interventions and equal effective case reproduction numbers) for England (red) and The Netherlands (blue). A) Proportion of hospitals with MRSA positive patients, arrows show the number of hospitals in both countries, showing faster dispersal in England as compared to the Netherlands. B) Mean MRSA prevalence among hospitals. C) The distribution of time to 50 hospitals infected. D) The percentage of simulated introductions of MRSA resulting in an epidemic.

Given the positive correlation between patient referrals and the incidence of hospital-acquired pathogens at local and regional level, the effect of referrals at national scale was investigated by comparing two countries with different referral patterns, England and the Netherlands. Referral networks were based on data from the NHS Hospital Episode Statistics and the Dutch National Medical Registry (figure 4, table S1). The spread of MRSA through the English hospital referral network was simulated as described previously [12], using the observed referral patterns for both countries. Equilibrium prevalence in England is reached faster than in the Netherlands (figure 4 A, B&C), the median time to infect 50 hospitals in the English network was 633 days, against 969 days in the Dutch network. Furthermore, in the English network significantly fewer simulations (0.7% vs 12.8%) ended in stochastic extinction (figure 4 D).

## Discussion

We reconstructed the hospital referral network for England by mapping all patient hospital admissions documented within one year onto a referral network and explored to what extend the position of hospitals within this referral network determines the hospital's incidence of typical hospital-acquired pathogens. The results show that patient referrals between hospitals correlate with hospital-specific MRSA rates as recorded by the English mandatory surveillance of MRSA bacteraemia. Generally, the incidence rate and connectedness tend to increase with hospital referral level and size. The size of a hospital serves here as an approximation of its function in the health care system; larger hospitals typically offer a wider range of treatments as they serve as specialist centres and have more referred admissions. We observed a consistent positive relation between the connectedness of hospitals, as measured by the IRI, and the incidence rate of MRSA bacteraemia. This is a clear indication that more connected hospitals have higher MRSA rates than less connected ones. A larger fraction of the patients admitted to the more connected hospitals is at risk of carrying a hospital-acquired infection, which results in a higher incidence. This correlation gets weaker as the mean MRSA bacteraemia incidence rate of all hospitals decreases, but it explains a significant part of the variance in the years 2001–2008.

Even when regarding each cluster as an independent network of hospitals, a positive correlation between connectedness and incidence can still be discerned, with most clusters showing a positive correlation between IRI and the observed incidence rate of MRSA bacteraemia. The fact that a number of positive correlations remained non-significant is likely due to the small size of clusters.

Between hospital regional clusters, MRSA rates differed significantly. Also, hospitals from larger clusters had, on average, a higher incidence than those from smaller clusters, indicating that a specific amount of colonisation pressure is exerted by other hospitals within the same cluster. The existence of regional differences in MRSA bacteraemia incidence rates can thus partly be explained by the network structure itself. Larger clusters have a higher chance of experiencing a founding event, such as a successful introduction followed by dispersal in one of the hospitals.

Infectious diseases have a higher probability of going extinct before reaching the entire population when the contact networks show a high degree of clustering or community structure [17]. The contact network of patients admitted to hospitals is hierarchically structured at multiple levels: first, patients have a higher probability of meeting patients on the same ward, then in the same hospital, followed by patients in hospitals in the same hospital cluster. This structure mitigates dispersal of hospital-acquired infections. Any differences in the health-care system affecting this modular structure of the hospital referral network will therefore inevitably have a bearing on the speed with which infections will spread.

Compared to the Dutch hospital referral network, the hospitals in the English network are more connected by the larger number of patients shared between them. This stronger connectivity makes the English network more permeable for hospital-acquired pathogens. An average English hospital shares on a yearly basis 4300 patients with other hospitals, whereas a Dutch hospital only shares about 1300 patients with other hospitals. At the same MRSA prevalence, an average English hospital will on a daily basis refer over three times more MRSA positive patients to other hospitals than a Dutch hospital. Although not all introductions will result in successful dispersal, the likelihood that this occurs is higher in English hospitals. This difference in permeability has important implications for infection control in hospitals. In England more stringent measures are required, whereas controlling the spread of hospital-acquired pathogens in the Netherlands is easier. The success of the Dutch ‘search and destroy’ policy may be assisted by a higher probability of stochastic extinction aborting nationwide outbreaks at an early stage.

Some imperfections in the data analysis need to be addressed. A relevant residual confounder which this study was unable to include is the medical condition of the patients. Acute conditions that require complex interventions are often the reason for referring patients to other hospitals while at the same time these patients may be more susceptible to hospital-acquired infections and have more exposure to antibiotics. To accurately correct for these effects, patient details that could not be extracted from the existing datasets need to be taken into account. The medical condition of the patients is the conventional explanation for the observed difference in HAIs rates between hospital categories. It can however not account for the observed correlation between size of the hospital regional cluster and MRSA bacteraemia rates and the observed regional heterogeneity in MRSA incidence in England. The burden of chronic disease as well as social deprivation scores in England have a known north-south gradient [18], [19] which did not coincide with average MRSA incidence rates ascertained for the different health-care collectives. Furthermore, the geographical clustering of MRSA clones in Europe [20] is a clear indication that hospital-acquired pathogens spread through regional hospital referral networks, rather than through the community. Likewise, patient referrals between regionally collaborating hospitals represents an analogy to patient transfers between wards, a known risk factor for HAIs in single hospitals [21], [22], quite likely describing the same phenomenon at a different scale.

Other studies have shown that hospital performance variables, such as bed occupancy, staff workload, high temporary nursing staff rates or low cleanliness scores are correlated with infection rates [5], [9]–[11]. It is not the aim of this study to challenge the value of these explanatory variables, rather we show that the strength of connectedness expressed as IRI has compelling explanatory power and consistently explains the variance in the reported MRSA bacteraemia rates for longer observation periods than performance variables used in previous investigations [5]. Whilst the conventional understanding of individual transmission events of hospital-acquired pathogens offers only partial explanatory power of the variance of MRSA rates in English hospitals, we have shown that a more comprehensive view of complex networks can be provided by the study of the interactions between hospitals. Improving performance variables has certainly its merits as can be seen by the recent success in reducing infection rates in England as a consequence of setting national targets. On admission screening, flagging and isolation of colonised patients reduces the collective case load of each regional cluster and with fewer cases percolating down the referral chain the correlation between IRI and MRSA incidence rate becomes predictably weaker as shown for FY 2008–09. National target setting should, however, be mindful of the relative position of hospitals within the regional referral network.

The general structure of hospital referral networks reveals a hierarchical modular network, that is shaped by the referral preferences and the speciality mix of individual hospitals, modified by multiple organisational decisions. Examples of other hierarchical modular networks can be found in biological and sociological contexts [23]–[28]. The ramifications of these findings should improve the ability to better understand and control the dispersal of hospital-acquired bacteria in the future. The network architecture of referral patterns provides an untapped potential for more efficient and cost effective regional infection policies. Concentrating resources earmarked for infection control on hospitals with the highest degree of connectivity would have a disproportionately larger effect than distributing the same resources haphazardly [29], [30]. The large teaching hospitals are an ideal target for intervention. Patients referred from these institutions are at highest risk of carrying a typical hospital-acquired pathogen. Efforts to screen these patients, either on discharge or upon admission in another hospital, will be more efficient than universal or random screening.

Another notion emerging from the hierarchical modular network architecture of referral patterns is that "rewiring" the connectivity between large and teaching hospitals from different regional clusters by introducing super-regional specialist centres would enhance the permeability and would facilitate a swifter nationwide dispersal through hospital networks. These specialist centres will act as hubs connecting clusters of hospitals, accelerating the national dissemination of HCAIs and ultimately stifling infection control.

## Materials and Methods

### Data

Annual data on individual hospital admissions in England from April 2006 to March 2007 was extracted from the NHS Hospital Episode Statistics (HES). Only data on health-care episodes in acute care hospitals was included, while data on admissions to primary care, mental health or single specialty trusts was excluded (see table S2 for a list of trusts included in this study).

Data on incidence rates of MRSA bacteraemia over the same period was retrieved for the included hospital trusts from the Department of Health's mandatory surveillance of MRSA bacteraemia, as publicly available from the HPA and DoH website [1], [2]. The MRSA bacteraemia incidence rate is measured as the number of cases per 10.000 bed days. Data over the longer period from April 2001 to March 2008 was also retrieved, to compare incidence rates of MRSA bacteraemia over multiple years.

### Network analysis

The English hospital referral network was reconstructed to determine the exposure of a hospital to patients that are admitted after a recent episode in another hospital. To that end, the per patient translocation probability (i.e. the probability that a patient translocates MRSA between the two hospitals), 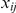, needed to be determined. This value is contingent on the probability of acquiring MRSA in the first hospital, 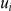, the probability of still carrying MRSA upon readmission, 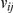, and the probability of spreading MRSA in the second hospital, 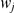.

Assuming that the prevalence of MRSA in the first hospital is at a constant low level, the probability of acquiring MRSA, 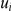 depends only on the length of stay of the patient, 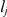, and can be expressed as 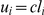, where 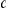 is the hazard of acquiring MRSA. For the receiving hospital, the probability that the same patient transmits MRSA, 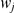, depends on the average per admission reproduction number 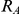 and the length of stay of the patient relative to the mean length of stay, 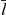, and can be expressed as 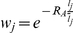.

The probability that a patient still carries MRSA upon readmission in turn depends on the time between discharge and subsequent admission 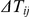 and the mean length of colonization 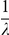, and can be expressed as 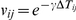. The product of the three probabilities then gives the per patient transfer probability of transmission from hospital 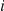 to hospital 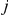, 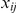.
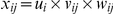



The sum over all patient transfers, 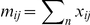 describes the entire hospital referral network 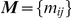. Each element 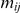 provides the total expected number of potential MRSA carriers referred from hospital 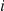 to hospital 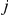.

### Infectious Relative Indegree

The total number of patients who were first admitted to any other hospital and then to hospital 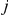, denoted by 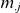, was calculated based on the expected number 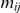 of each of the referring hospitals. These patients could carry MRSA and have the potential to introduce it in hospital 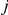.

With the total number of admitted patients to hospital 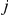 over the period of interest, 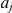, the probability that a patient admitted to hospital j carries the relevant pathogen acquired during a recent health-care episode in another hospital can be calculated as 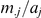, coined “infectious relative indegre” of hospital 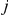 (IRI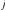). This variable IRI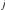 provides a measure of the relative exposure of a hospital 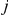 to patients that are admitted after a recent episode in another hospital.

### Hospital regional clusters

To expose the structure of the English hospital referral network, the hospitals were grouped into hospital regional clusters, based on the weights of the connections between all hospitals, 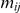, using a community detection algorithm [16] which searches for division of the network with the highest modularity 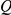 [31]. This network modularity is defined [16], [31] as 
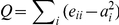
 where 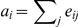 and 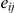 is the total weight of the connections between cluster 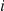 and 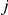. The modularity 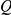 has a maximum of 1, indicating a very strong community structure, whereas 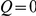 indicates a random structure.

The robustness of the clusters needed to be assessed, because the weights of the connections between hospitals in 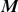 are based on the patient referrals of a single observational period, the financial year 2006–2007, and small errors may occur especially along the weak connections. To that end, a large set of ‘bootstrap’ networks was simulated.

For each bootstrap network, a new number of patients, 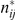, exchanged between hospital 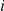 and 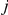 was drawn from a Poisson distribution with 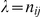, where 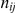 is the actual reported number of exchanged patients. The ‘bootstrap’ connectedness between the hospitals was calculated as 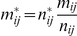, where 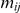 is the actual reported connectedness between 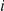 and 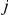. The community structure algorithm was applied for 5000 ‘bootstrap’ networks and bootstrap values, 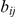, were calculated for each possible pair of hospitals 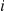 and 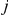, as the fraction of ‘bootstrap’ networks where they were clustered in the same group. The mean bootstrap value for the clusters of hospitals was calculated as 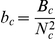 where 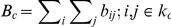 denotes the sum over the bootstrap values in cluster 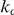 and 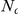 denotes the number of hospitals in collective 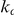. Subsequently, the partitioning with the highest mean 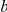 was assigned as the optimal cluster assignment.

The measure for the weighted connections between hospitals, 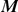, is a simplification of the true underlying contact network between patients. Despite the fact that the weights and direction of the connections are retained in the measured IRI for each hospital, it only gives an approximation of the risk of introduction of a hospital-acquired infection. However, the IRI does capture the network disparity between hospitals in an effective way, and explains the observed differences in rates of hospital-acquired infections between hospitals sufficiently well.

### Statistical analysis

The partial correlation coefficient between the incidence rates of MRSA bacteraemia reported for single hospitals and the logarithm of the IRI of that hospital was measured as an estimate of the effect of patient referrals on the incidence of infections caused by hospital-acquired bacteria. The logarithm of the IRI was used, to account for saturation effects (see figure S2). Further, the partial correlation coefficient, controlling for the hospital regional clusters, was used as it measures the standard (product-moment) correlation between incidence and the predictor variable (here log IRI) while taking the mean incidence rate and mean IRI of the hospital's regional cluster into account (See figure S3).

### Agent-based model

The dispersal dynamics within the English and the Dutch hospital referral network [12] was compared at national level, using an agent-based model approach. First, for both the Netherlands and England the distribution of length of stay, time between admissions and the number of changes between hospitals of patients, stratified by their number of admissions was measured. Furthermore, the distribution of number of times patients were admitted to hospital, and the number of admissions per hospital were measured. Subsequently, a simulated dataset was created for both countries by resampling from these distributions. To allow for an unbiased comparison, two parameters (length of stay and readmission probability) were adjusted to maintain an equal per admission case reproduction number [32] for both networks.

All patients were tracked as they were admitted to the hospitals in the simulated dataset. They can either be susceptible or colonized, and colonized (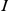) individuals transmit the pathogen to susceptible (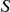) individuals within the same hospital on the same day with rate 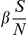, where 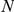 denotes the total number of individuals in the hospital. No assumptions were made about the within-hospital structure, and mass action mixing assumed within each hospital.

Each simulation was started by colonizing 5% of the patients of a single hospital in the network, subsequently using each hospital as the starting point. Dispersal was measured by tracking colonized patients, referrals to other hospitals and transmission events for every day in all hospitals for a period of 15 years. The results of a typical simulation are depicted in movie S1. To determine the mean prevalence, the number of hospitals infected, and the proportion of stochastic extinctions, each experiment consisted of a minimum of 5,000 simulations.

## Supporting Information

Figure S1 **The network properties of the English hospital referral network, describing the distribution of connection weights, 5% strongest links, clustering coefficient and connection disassortativeness.**
(PDF)

Figure S2 **The relation between Infectious Relative Indegree (IRI) and model MRSA equilibrium prevalence, showing both linear and log transformed IRI.**
(PDF)

Figure S3 **The relation between IRI and observed MRSA bacteraemia incidence, over all 8 years of data from the mandatory MRSA bacteraemia surveillance.**
(PDF)

Movie S1 **Shows one realisation of the 5,000 model simulations.** Main panel shows the geographical distribution of the hospital trusts in England. The animation captures the dispersal dynamics of hospital-acquired bacteria after an outbreak in a single hospital in the East Midlands whereby 5% of all patients have been infected. Yellow radiating lines indicate transfer of infected patients to other hospitals. The colour of the placemarks indicates the prevalence in individual hospital (Black, hospitals that never encountered infected patients. Gray, hospitals with history of infected patients but no present cases. Pale yellow, hospitals with 0–10% present cases. Yellow, hospitals with 10–15% present cases. Red, hospitals with >15% present). The top right panel shows the point prevalence in each of the hospitals as time progresses, the middle right panel shows the percentage of hospitals that have encountered the infection (blue line) and the percentage of hospitals that have present cases. The lover right panel indicates the progression in time.
(AVI)

Table S1 **Characteristics of the hospital referral networks in England and the Netherlands, based on data from respectively the NHS Hospital Episode Statistics and the Dutch National Medical Registry.**
(PDF)

Table S2 **Overview of the included hospital trusts.**
(PDF)

Text S1 **Description of the analyses of the properties of the English patient referral network, including the calculation of the clustering coefficient, degree and neighbour degree as a measure for the disassortativeness of the connections between hospitals.**
(PDF)

## References

[1] Health Protection Agency: Mandatory Surveillance of Staphylococcus aureus bacteraemia.. http://www.hpa.org.uk/web/HPAweb&Page&HPAwebutoListName/Page/1191942169773.

[2] Department of Health website. MRSA surveillance system: Results.. http://www.dh.gov.uk/en/Publicationsandstatistics/Publications/Publications.

[3] Harbarth S, Martin Y, Rohner P, Henry N, Auckenthaler R (2000). Effect of delayed infection control measures on a hospital outbreak of methicillin-resistant Staphylococcus aureus.. Journal of Hospital Infection.

[4] Higgins A, Lynch M, Gethin G (2010). Can ‘search and destroy’ reduce nosocomial methicillinresistant Staphylococcus aureus in an Irish hospital?. The Journal of hospital infection.

[5] Department of Health (2007). Hospital organisation, specialty mix and MRSA..

[6] Baquero F (2004). From pieces to patterns: evolutionary engineering in bacterial pathogens.. Nature reviews Microbiology.

[7] Enright MC, Robinson DA, Randle G, Feil EJ, Grundmann H (2002). The evolutionary history of methicillin-resistant Staphylococcus aureus (MRSA).. Proceedings of the National Academy of Sciences of the United States of America.

[8] Ansari F, Erntell M, Goossens H, Davey P (2009). The European surveillance of antimicrobial consumption (ESAC) point-prevalence survey of antibacterial use in 20 European hospitals in 2006.. Clinical infectious diseases.

[9] Halwani M, Solaymani-Dodaran M, Grundmann H, Coupland C, Slack R (2006). Cross-transmission of nosocomial pathogens in an adult intensive care unit: incidence and risk factors.. The Journal of hospital infection.

[10] Cunningham JB, Kernohan WG, Sowney R (2005). Bed occupancy and turnover interval as determinant factors in MRSA infections in acute settings in Northern Ireland: 1 April 2001 to 31 March 2003.. The Journal of hospital infection.

[11] Vicca AF (1999). Nursing staff workload as a determinant of methicillin-resistant Staphylococcus aureus spread in an adult intensive therapy unit.. The Journal of hospital infection.

[12] Donker T, Wallinga J, Grundmann H (2010). Patient referral patterns and the spread of hospitalacquired infections through national health care networks.. PLoS computational biology.

[13] Huang SS, Avery TR, Song Y, Elkins KR, Nguyen CC (2010). Quantifying interhospital patient sharing as a mechanism for infectious disease spread.. Infection control and hospital epidemiology.

[14] Iwashyna TJ, Christie J, Moody J, Kahn J, Asch D (2009). The structure of critical care transfer networks.. Medical care.

[15] Robotham JV, Scarff CA, Jenkins DR, Medley GF (2007). Meticillin-resistant Staphylococcus aureus (MRSA) in hospitals and the community: model predictions based on the UK situation.. Journal of Hospital Infection.

[16] Clauset A (2005). Finding local community structure in networks.. Physical Review E – Statistical, Nonlinear, and Soft Matter Physics.

[17] Salathé M, Jones JH (2010). Dynamics and Control of Diseases in Networks with Community Structure.. PLoS Computational Biology.

[18] Raleigh VS, Kiri VA (1997). Life expectancy in England: variations and trends by gender, health authority, and level of deprivation.. Journal of Epidemiology & Community Health.

[19] Woods L, Rachet B, Riga M, Stone N, Shah A (2005). Geographical variation in life expectancy at birth in England and Wales is largely explained by deprivation.. Journal of Epidemiology & Community Health.

[20] Grundmann H, Aanensen DM, van den Wijngaard CC, Spratt BG, Harmsen D (2010). Geographic distribution of Staphylococcus aureus causing invasive infections in Europe: a molecularepidemiological analysis.. PLoS medicine.

[21] Dziekan G, Hahn A, Thüne K, Schwarzer G, Schäfer K (2000). Methicillin-resistant Staphylococcus aureus in a teaching hospital: investigation of nosocomial transmission using a matched case-control study.. The Journal of hospital infection.

[22] Eveillard M, Quenon JL, Rufat P, Mangeol A, Fauvelle F (2001). Association between hospitalacquired infections and patients' transfers.. Infection control and hospital epidemiology.

[23] Natarajan M (2006). Understanding the Structure of a Large Heroin Distribution Network: A Quantitative Analysis of Qualitative Data.. Journal of Quantitative Criminology.

[24] Newman MEJ (2003). The structure and function of complex networks.. SIAM Review.

[25] Strogatz SH (2001). Exploring complex networks.. Nature.

[26] Ravasz E, Somera AL, Mongru DA, Oltvai ZN, Barabási AL (2002). Hierarchical organization of modularity in metabolic networks.. Science (New York, NY).

[27] Anderson TK, Sukhdeo MVK (2011). Host Centrality in Food Web Networks Determines Parasite Diversity.. PLoS ONE.

[28] Hao D, Li C (2011). The dichotomy in degree correlation of biological networks.. PloS one.

[29] Albert R, Jeong H, Barabasi AL (2000). Error and attack tolerance of complex networks.. Nature.

[30] Karkada UH, Adamic LA, Kahn JM, Iwashyna TJ (2011). Limiting the spread of highly resistant hospital-acquired microorganisms via critical care transfers: a simulation study.. Intensive care medicine.

[31] Girvan M, Newman MEJ (2002). Community structure in social and biological networks.. Proceedings of the National Academy of Sciences of the United States of America.

[32] Cooper BS, Medley GF, Stone SP, Kibbler CC, Cookson BD (2004). Methicillin-resistant Staphylococcus aureus in hospitals and the community: Stealth dynamics and control catastrophes.. Proceedings of the National Academy of Sciences of the United States of America.

