# Supplementary material for: Hospital Networks and the Dispersal of Hospital-Acquired Pathogens by Patient Transfer
Source: PLoS One. 2012 Apr 25;7(4):e35002. doi: 10.1371/journal.pone.0035002 (PMC3338821; doi:10.1371/journal.pone.0035002)
Supplement: Figure S1 — The network properties of the English hospital referral network, describing the distribution of connection weights, 5% strongest links, clustering coefficient and connection disassortativeness. (PDF) [file pone.0035002.s001.pdf]

Network structure

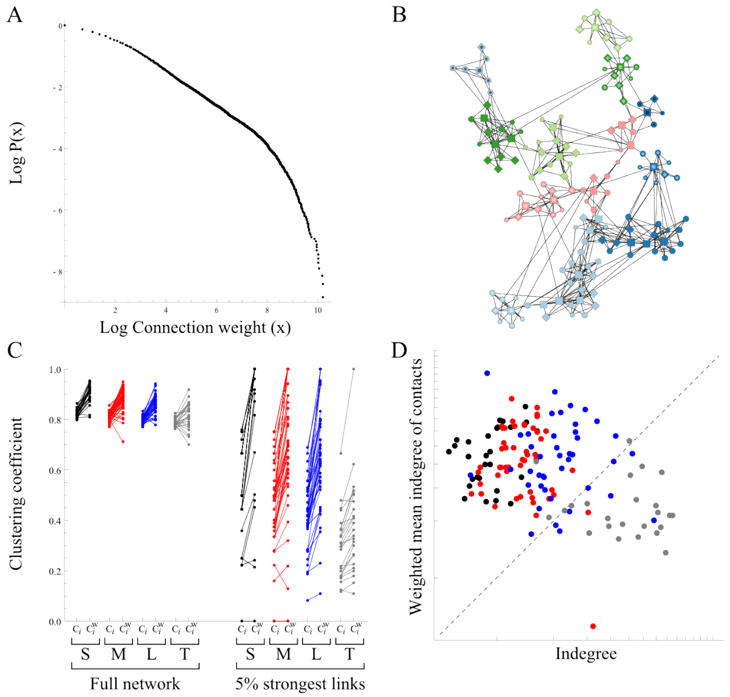

Figure : The network properties of the English hospital referral network. A) The cumulative density function $P(x)$ [1] of the weights of the connections between hospitals, showing the fraction of links with a weight equal or lower than a certain link strength x. B) The 5% strongest connections in the network contribute to 84% of the total weights of the connections in the network. Despite removing 95% of the links, all hospitals remain connected in the network. C) The unweighted (C_i) and weighted (C_i^w) clustering coefficient per hospital, stratified by hospital type and for both the complete and 5% stongest links network. D) Hospitals with a high degree of connectedness tend to be connected to hospitals with lower degrees, and vice-versa.

References

[1] Clauset A, Shalizi C, Newman MEJ. Power-law distributions in empirical data. SIAM Review. 2009;51(4):661–703.
