# Supplementary material for: Hospital Networks and the Dispersal of Hospital-Acquired Pathogens by Patient Transfer
Source: PLoS One. 2012 Apr 25;7(4):e35002. doi: 10.1371/journal.pone.0035002 (PMC3338821; doi:10.1371/journal.pone.0035002)
Supplement: Figure S2 — The relation between Infectious Relative Indegree (IRI) and model MRSA equilibrium prevalence, showing both linear and log transformed IRI. (PDF) [file pone.0035002.s002.pdf]

Saturation of Infectious Relative Indegree (IRI)

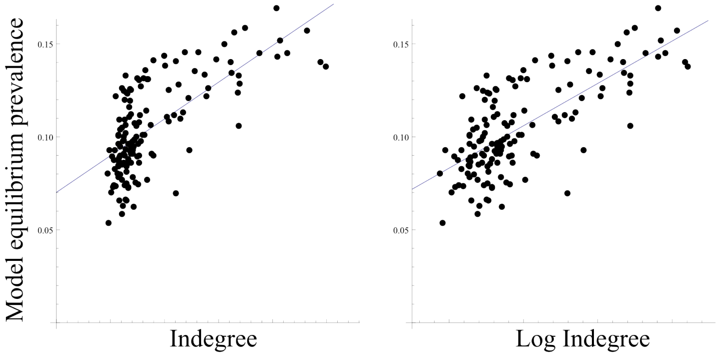

Figure : The relation between IRI and model MRSA equilibrium prevalence, plotted using A) IRI and B) Log IRI. The prevalence shows a stronger positive linear correlation with log IRI than IRI.
