# Supplementary material for: Hospital Networks and the Dispersal of Hospital-Acquired Pathogens by Patient Transfer
Source: PLoS One. 2012 Apr 25;7(4):e35002. doi: 10.1371/journal.pone.0035002 (PMC3338821; doi:10.1371/journal.pone.0035002)
Supplement: Figure S3 — The relation between IRI and observed MRSA bacteraemia incidence, over all 8 years of data from the mandatory MRSA bacteraemia surveillance. (PDF) [file pone.0035002.s003.pdf]

Correlation IRI and MRSA bacteraemia

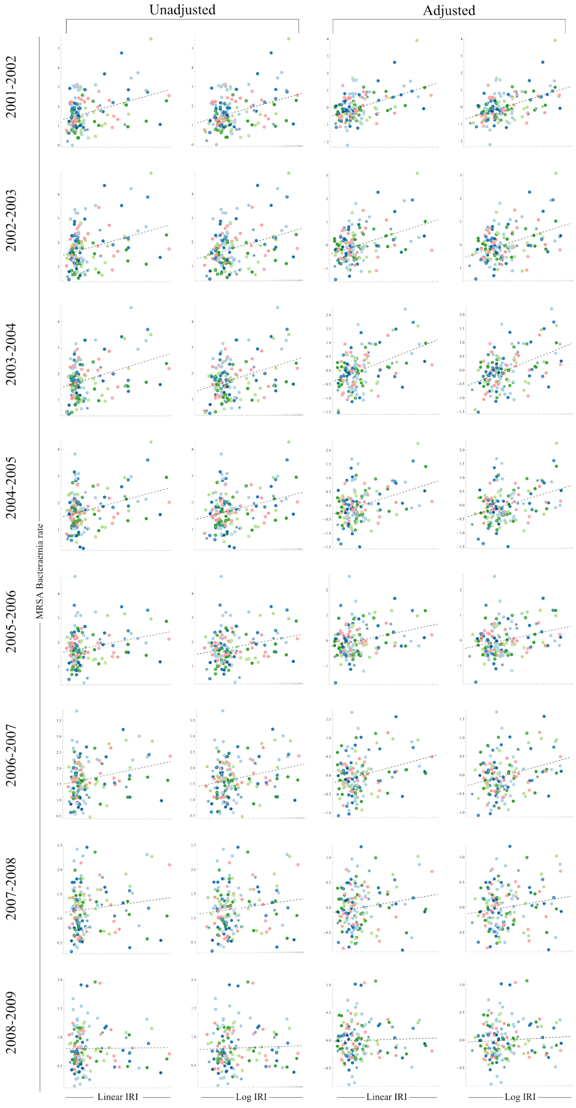

Figure : The relation between IRI and observed MRSA bacteraemia incidence, over all 8 years of data. The left two columns show the data unadjusted for the hospital clusters, the right to columns show the adjusted data, with the mean MRSA rate and mean IRI for each cluster subtracted, as used for the calculation of the partial correlation coefficient.
