# Supplementary material for: Hospital Networks and the Dispersal of Hospital-Acquired Pathogens by Patient Transfer
Source: PLoS One. 2012 Apr 25;7(4):e35002. doi: 10.1371/journal.pone.0035002 (PMC3338821; doi:10.1371/journal.pone.0035002)
Supplement: Table S1 — Characteristics of the hospital referral networks in England and the Netherlands, based on data from respectively the NHS Hospital Episode Statistics and the Dutch National Medical Registry. (PDF) [file pone.0035002.s005.pdf]

# Properties health care system England & the Netherlands

Table : Characteristics of the hospital referral networks in England and the Netherlands, based on data from respectively the NHS Hospital Episode Statistics (from April 2006 until March 2007) and the National Medical Registry (from January 2004 until December 2004).

| | England | The Netherlands |
|---|---|---|
| Inhabitants | 51.092.000 | 16.282.000 |
| Patients | 7.420.219 | 1.676.704 |
| Admissions | 12.929.171 | 2.611.452 |
| Patients per capita (/1.000) | 145 | 103 |
| Admissions per capita (/1.000) | 253 | 160 |
| Hospital organizations | 146 | 98 |
| Readmissions to same hospital | 4.891.343 | 805.128 |
| Readmissions per hospital | 33502,35 | 8215,59 |
| Between hospital referrals | 617.609 | 129.620 |
| Between hospital referrals per hospital | 4.230 | 1.322 |
| Network Modularity Q[1] | 0,72 | 0,57 |
| Network Modularity Q incl. self-referral[1] | 0,88 | 0,78 |

# References


[1] Newman MEJ, Girvan M. Finding and evaluating community structure in networks. Physical Review E - Statistical, Nonlinear and Soft Matter Physics. 2004;69(2 Pt 2):16.


[1] The network modularity Q [1] was first calculated based on between hospital transferrals, and then recalculated to take readmissions to the same hospital into account.
