# Supplementary material for: Hospital Networks and the Dispersal of Hospital-Acquired Pathogens by Patient Transfer
Source: PLoS One. 2012 Apr 25;7(4):e35002. doi: 10.1371/journal.pone.0035002 (PMC3338821; doi:10.1371/journal.pone.0035002)
Supplement: Table S2 — Overview of the included hospital trusts. (PDF) [file pone.0035002.s006.pdf]

Hospital Trusts

Table : Hospital trusts used. Cluster number reflects numbers as indicated in figure 1

Nr.	Code	Trust Name	Category	Trust SHA	Cluster	Admissions
1	RA2	Royal Surrey County Hospital	M	South East	1	56063
2	RA3	Weston Area Health	S	South West	6	28761
3	RA4	Yeovil District Hospital	S	South West	6	31364
4	RA7	University Hospitals Bristol	T	South West	6	121466
5	RA9	South Devon Healthcare	M	South West	6	57310
6	RAE	Bradford Teaching Hospitals	L	Yorkshire & the Humber	7	102166
7	RAJ	Southend University Hospital	M	East of England	2	84357
8	RAL	Royal Free Hampstead	T	London	2	80724
9	RAP	North Middlesex University Hospital	M	London	2	50568
10	RAS	Hillingdon Hospital	M	London	1	54128
11	RAX	Kingston Hospital	M	London	1	58584
12	RBA	Taunton & Somerset	M	South West	6	73769
13	RBD	Dorset County Hospital	S	South West	10	49175
14	RBK	Walsall Hospitals	M	West Midlands	3	63079
15	RBL	Wirral University Teaching Hospital	L	North West	11	92899
16	RBN	St Helens & Knowsley Hospitals	M	North West	11	65597
17	RBT	Mid Cheshire Hospitals	S	North West	3	55641

Nr.	Code	Trust Name	Category	Trust SHA	Cluster	Admissions
18	RBZ	Northern Devon Healthcare	S	South West	6	37320
19	RC1	Bedford Hospital	S	East of England	9	42829
20	RC3	Ealing Hospital	S	London	1	38290
21	RC9	Luton & Dunstable Hospital	M	East of England	2	61608
22	RCB	York Hospitals	M	Yorkshire & the Humber	7	71054
23	RCC	Scarborough & North East Yorkshire Health Care	M	Yorkshire & the Humber	7	38531
24	RCD	Harrogate & District	S	Yorkshire & the Humber	7	38730
25	RCF	Airedale	S	Yorkshire & the Humber	7	48827
26	RCX	The Queen Elizabeth Hospital King's Lynn	S	East of England	9	58337
27	RD1	Royal United Hospital Bath	M	South West	6	60819
28	RD3	Poole Hospital	M	South West	10	69009
29	RD7	Heatherwood & Wexham Park Hospitals	M	South East	5	80535
30	RD8	Milton Keynes Hospital	S	South East	5	31831
31	RDD	Basildon & Thurrock University Hospitals	M	East of England	2	83634
32	RDE	Colchester Hospital University	M	East of England	2	68371
33	RDU	Frimley Park Hospital	M	South East	1	68303
34	RDZ	The Royal Bournemouth & Christchurch Hospitals	M	South West	10	87326
35	RE9	South Tyneside	S	North East	8	33066
36	REF	Royal Cornwall Hospitals	L	South West	6	124913
37	REM	Aintree University Hospitals	M	North West	11	85953
38	RF4	Barking, Havering & Redbridge Hospitals	L	London	2	130882
39	RFF	Barnsley Hospital	S	Yorkshire & the Humber	12	54830
40	RFR	The Rotherham	M	Yorkshire & the Humber	12	66349
41	RFS	Chesterfield Royal Hospital	M	East Midlands	12	65223
42	RFW	West Middlesex University Hospital	S	London	1	20799
43	RGC	Whipps Cross University Hospital	M	London	2	69958
44	RGN	Peterborough & Stamford Hospitals	M	East of England	9	67480

Nr.	Code	Trust Name	Category	Trust SHA	Cluster	Admissions
45	RGP	James Paget University Hospitals	M	East of England	9	63310
46	RGQ	Ipswich Hospital	M	East of England	9	76966
47	RGR	West Suffolk Hospitals	S	East of England	9	49107
48	RGT	Cambridge University Hospitals	T	East of England	9	150975
49	RH8	Royal Devon & Exeter	L	South West	6	113310
50	RHM	Southampton University Hospitals	T	South East	10	110054
51	RHQ	Sheffield Teaching Hospitals	T	Yorkshire & the Humber	12	260704
52	RHU	Portsmouth Hospitals	L	South East	10	130742
53	RHW	Royal Berkshire	L	South East	5	114874
54	RJ1	Guy's & St. Thomas'	T	London	1	153618
55	RJ2	The Lewisham Hospital	M	London	1	54806
56	RJ6	Mayday Healthcare	M	London	1	60955
57	RJ7	St. George's Healthcare	T	London	1	115527
58	RJC	South Warwickshire General Hospitals	S	West Midlands	3	44608
59	RJD	Mid Staffordshire	S	West Midlands	3	52927
60	RJE	University Hospital of North Staffordshire	L	West Midlands	3	166574
61	RJF	Burton Hospitals	S	West Midlands	5	45393
62	RJL	Northern Lincolnshire & Goole Hospitals	L	Yorkshire & the Humber	7	96364
63	RJN	East Cheshire	S	North West	4	28859
64	RJR	Countess of Chester Hospital	M	North West	11	75579
65	RJZ	King's College Hospital	T	London	1	88813
66	RK5	Sherwood Forest Hospitals	M	East Midlands	5	71543
67	RK9	Plymouth Hospitals	T	South West	6	109390
68	RKB	University Hospitals Coventry & Warwickshire	T	West Midlands	3	100299
69	RKE	The Whittington Hospital	M	London	2	42682
70	RL4	Royal Wolverhampton Hospitals	L	West Midlands	3	96866
71	RLN	City Hospitals Sunderland	L	North East	8	89824

Nr.	Code	Trust Name	Category	Trust SHA	Cluster	Admissions
72	RLQ	Hereford Hospitals	S	West Midlands	3	34857
73	RLT	George Eliot Hospital	S	West Midlands	3	41692
74	RM1	Norfolk & Norwich University Hospitals	L	East of England	9	160455
75	RM2	University Hospital of South Manchester	T	North West	4	72959
76	RM3	Salford Royal	T	North West	4	96383
77	RM4	Trafford Healthcare	S	North West	4	28881
78	RMC	Royal Bolton Hospital	M	North West	4	69824
79	RMP	Tameside Hospital	S	North West	4	44319
80	RN1	Winchester & Eastleigh Healthcare	S	South East	10	36572
81	RN3	Great Western Hospitals	M	South West	5	64219
82	RN5	Basingstoke & North Hampshire	M	South East	10	38941
83	RN7	Dartford & Gravesham	S	South East	1	40627
84	RNA	The Dudley Group of Hospitals	M	West Midlands	3	99509
85	RNH	Newham University Hospital	M	London	2	54009
86	RNJ	Barts & the London	T	London	2	99824
87	RNL	North Cumbria University Hospitals	M	North West	8	85847
88	RNQ	Kettering General Hospital	S	East Midlands	5	67777
89	RNS	Northampton General Hospital	M	East Midlands	5	83453
90	RNZ	Salisbury	S	South West	10	51545
91	RP5	Doncaster & Bassetlaw Hospitals	L	Yorkshire & the Humber	12	106256
92	RPA	Medway	M	South East	1	58553
93	RQ6	Royal Liverpool & Broadgreen University Hospitals	T	North West	11	74960
94	RQ8	Mid Essex Hospital Services	M	East of England	2	67083
95	RQM	Chelsea & Westminster Hospital	T	London	1	66906
96	RQQ	Hinchingbrooke Healthcare	S	East of England	9	36172
97	RQW	Princess Alexandra Hospital	M	East of England	2	50670
98	RQX	Homerton University Hospital	S	London	2	49701

Nr.	Code	Trust Name	Category	Trust SHA	Cluster	Admissions
99	RR1	Heart of England	L	West Midlands	3	239551
100	RR7	Gateshead Health	M	North East	8	41778
101	RR8	Leeds Teaching Hospitals	T	Yorkshire & the Humber	7	208135
102	RRF	Wrightington, Wigan & Leigh	L	North West	4	78951
103	RRK	University Hospital Birmingham	T	West Midlands	3	109995
104	RRV	University College London Hospitals	T	London	2	41564
105	RTD	The Newcastle upon Tyne Hospitals	T	North East	8	178525
106	RTE	Gloucestershire Hospitals	L	South West	6	156241
107	RTF	Northumbria Healthcare	L	North East	8	107668
108	RTG	Derby Hospitals	L	East Midlands	5	127219
109	RTH	Oxford Radcliffe Hospitals	T	South East	5	172046
110	RTK	Ashford & St Peter's Hospitals	M	South East	1	59776
111	RTP	Surrey & Sussex Healthcare	M	South East	1	63825
112	RTR	South Tees Hospitals	L	North East	8	139816
113	RTX	University Hospitals of Morecambe Bay	L	North West	4	88709
114	RV8	North West London Hospitals	L	London	1	91193
115	RVJ	North Bristol	L	South West	6	176137
116	RVL	Barnet & Chase Farm Hospitals	L	London	2	93104
117	RVR	Epsom & St Helier University Hospitals	L	London	1	125016
118	RVV	East Kent Hospitals University	L	South East	1	126361
119	RVW	North Tees & Hartlepool	M	North East	8	79246
120	RVY	Southport & Ormskirk Hospital	M	North West	11	51790
121	RW3	Central Manchester University Hospitals	T	North West	4	149940
122	RW6	Pennine Acute Hospitals	L	North West	4	211568
123	RWA	Hull & East Yorkshire Hospitals	L	Yorkshire & the Humber	7	137201
124	RWD	United Lincolnshire Hospitals	L	East Midlands	5	135035
125	RWE	University Hospitals of Leicester	T	East Midlands	5	218443

Nr.	Code	Trust Name	Category	Trust SHA	Cluster	Admissions
126	RWF	Maidstone & Tunbridge Wells	L	South East	1	96326
127	RWG	West Hertfordshire Hospitals	L	East of England	2	76857
128	RWH	East & North Hertfordshire	L	East of England	2	73025
129	RWJ	Stockport	M	North West	4	74681
130	RWP	Worcestershire Acute Hospitals	L	West Midlands	3	93068
131	RWW	Warrington & Halton Hospitals	M	North West	11	66038
132	RWY	Calderdale & Huddersfield	L	Yorkshire & the Humber	7	89951
133	RX1	Nottingham University Hospitals	T	East Midlands	5	194692
134	RXC	East Sussex Hospitals	L	South East	1	99943
135	RXF	Mid Yorkshire Hospitals	L	Yorkshire & the Humber	7	128300
136	RXH	Brighton & Sussex University Hospitals	T	South East	1	95290
137	RXK	Sandwell & West Birmingham Hospitals	L	West Midlands	3	119706
138	RXL	Blackpool, Fylde & Wyre Hospitals	L	North West	4	98587
139	RXN	Lancashire Teaching Hospitals	L	North West	4	179456
140	RXP	County Durham & Darlington	L	North East	8	105233
141	RXQ	Buckinghamshire Hospitals	M	South East	5	89822
142	RXR	East Lancashire Hospitals	L	North West	4	132880
143	RXW	Shrewsbury & Telford Hospital	L	West Midlands	3	102022
144	RYJ	Imperial College Healthcare	T	London	1	164215
145	RYQ	South London Healthcare	L	London	1	161069
146	RYR	Western Sussex Hospitals	M	South East	1	102057
