# Supplementary material for: Hospital Networks and the Dispersal of Hospital-Acquired Pathogens by Patient Transfer
Source: PLoS One. 2012 Apr 25;7(4):e35002. doi: 10.1371/journal.pone.0035002 (PMC3338821; doi:10.1371/journal.pone.0035002)
Supplement: Text S1 — Description of the analyses of the properties of the English patient referral network, including the calculation of the clustering coefficient, degree and neighbour degree as a measure for the disassortativeness of the connections between hospitals. (PDF) [file pone.0035002.s007.pdf]

Network properties

To explore the position of individual hospitals within the patient referral network, we calculated the cluster coefficients [1, 2] for all hospitals. This can be done with or without the use of information about the connection weights. Without information about the weight of the connections, the cluster coefficient, c_i, measures the fraction of neighbours of one node (in this case a hospital), that are connected. This coefficient can be transformed to include edge weights when they are between 0 and 1 [3]. We therefore normalized the edge weights by dividing by the maximum weight. However, the English hospital referral network is highly interconnected, with each hospital sharing patients with on average 77% of the other hospitals. As a consequence, a large part of the triplets form triangles (See figure S3C). The large teaching hospitals have lower clustering coefficients than the general hospitals (figure S3C). Repeating the analysis with only the strongest 5% of the connections, that account for 84% of the total weight of all the connections in the network, gave the same result. This indicates that the surrounding general hospitals are connected strongly with the central teaching hospital, and weakly with other teaching hospitals.

To quantify the extend in which large teaching hospitals connect with smaller hospitals, we determined the correlation between the degree of a hospital and the weighted average degree of its neighbours. The weighted average degree of the neighbours of hospital i is defined[3] as

$$k^w_{nn,i} = \frac{1}{s} \sum_{j=1}^{N} m_{ij} k_j,$$

where m_{ij} is the connection weight, the total weight of the connections of hospital i is $s_i = \sum_j m_{ij}$ and a neighbours degree is $k_j = \sum_i m_{ij}$. The negative correlation (r=-0.34) between the hospitals and their neighbours indegrees (figure S3D) clearly shows disassortative mixing between hospitals: highly connected hospitals tend to connect with less connected ones. This disassortative mixing and the low clustering coefficient of highly connected teaching hospitals, are clear indications of a modular network with local hierarchy.

References

[1] Watts DJ, Strogatz SH. Collective dynamics of 'small-world' networks. Nature. 1998 Jun;393(6684):440–2.

[2] Newman MEJ, Strogatz SH, Watts DJ. Random graphs with arbitrary degree distributions and their applications. Physical Review E. 2001 Jul;64(2):1–17.

[3] Barrat A, Barthélemy M, Pastor-Satorras R, Vespignani A. The architecture of complex weighted networks. Proceedings of the National Academy of Sciences of the United States of America. 2004 Mar;101(11):3747–52.
